# Comment on: Classification of plastic surgery malpractice complaints brought before the São Paulo Medical Board that were treated as professional-misconduct cases: a cross-sectional study

**DOI:** 10.1590/1516-3180.2020.0263.08092020

**Published:** 2020-10-26

**Authors:** Thiago Gonçalves dos Santos Martins

**Affiliations:** I MD. Doctoral Student, Universidade Federal de São Paulo (SP), São Paulo (SP), Brazil, and Doctoral Student, Universidade de Coimbra (UC), Coimbra, Portugal.

Dear Editor,

In response to the article titled “Classification of plastic surgery malpractice complaints brought before the São Paulo Medical Board that were treated as professional-misconduct cases: a cross-sectional study” published in your esteemed journal, which is a well-thought-out and well-written paper, I would like to raise a few points regarding this study.

The article reported that the number of complaints lodged decreased over the last two years reviewed, although complaints regarding malpractice and poor doctor-patient relationships increased by 10% over the same period.^1^

The specialties with the greatest number of lawsuits in the United States are gynecology/obstetrics, general surgery and internal medicine.^2^ The factors that may explain the increasing numbers of lawsuits include the population's greater knowledge about their rights and the influence of the media. Deterioration in the quality of the doctor-patient relationship has contributed to this situation.^3^ Medical schools are focusing on training technical professionals, which thus reduces the teaching time available for bioethics.^4^ Teaching of ethics has a dual function. The first is to improve students' capacity for bioethical reflection and the second is to shape them into citizens who are aware of the importance of their profession within society.

To avoid medical malpractice, improvement of the doctor-patient relationship and communication between the doctor and the family must be emphasized, in addition to encouraging proper filling out of medical records. Currently, technology occupies a large space within medical care and has replaced important moments for anamnesis and physical examination, which are essential factors that form part of Hippocratic medical practice. The medical curriculum needs to be complemented with lectures, online courses and direct exchanges of experiences between professionals, thereby providing greater exposure to ethical content and improving learning.

Thus, it is important to invest in prevention of bad practices, so as to train medical professionals who have greater commitment to good medical practice.
